# How do peer group reflection meetings support medical students’ learning and personal development during clinical rotations?

**DOI:** 10.1186/s12909-023-04481-0

**Published:** 2023-07-06

**Authors:** Valerie van den Eertwegh, Renée E. Stalmeijer

**Affiliations:** 1 Researcher and Trainer in Communication and Behavioral Change Programs at the Skillslab Department, Faculty of Health, Medicine and Life Sciences, Maastricht University, Universiteitssingel 5, Maastricht, 6229 ES the Netherlands; 2 School of Health Professions Education and Chair of Taskforce Program Evaluation, Department of Educational Development and Research, Faculty of Health, Medicine and Life Sciences, Maastricht University, Maastricht, The Netherlands

**Keywords:** Intervision Meetings, Reflective group method, Well-being, Coping, Clinical rotations

## Abstract

**Background:**

Medical schools look to support students in coping with challenges and stressors related to clinical rotations. One potential approach is implementing Intervision Meetings (IM): a peer group reflection method during which students address challenging situations and personal development issues with peers, guided by a coach. Its implementation and perceived effectiveness in undergraduate medical education has however not yet been widely studied and described. This study evaluates how students perceive the effect of a three-year IM-programme during their clinical rotations, and explores which processes and specific factors support students’ personal development and learning during clinical rotations.

**Methods:**

Using an explanatory Mixed Methodology, medical students participating in IM were asked to evaluate their experiences through a questionnaire at three time points. Questionnaire results were further explored through three focus groups. Data were analysed using descriptive statistics and thematic analysis.

**Results:**

Three hundred fifty seven questionnaires were filled out by students across the three time points. Students perceived IM to contribute to their ability to cope with challenging situations during clinical rotations. Participants in the focus groups described how IM created an increase in self-awareness by active self-reflection supported by peers and the coach. Sharing and recognizing each other’s’ situations, stories or problems; as well as hearing alternative ways of coping, helped students to put things into perspective and try out alternative ways of thinking or behaving.

**Conclusions:**

IM can help students to better deal with stressors during clinical rotations and approach challenges as learning opportunities under the right circumstances. It is a potential method medical schools can use to aid their students on their journey of personal and professional development.

**Supplementary Information:**

The online version contains supplementary material available at 10.1186/s12909-023-04481-0.

## Introduction

Addressing medical student’s emotional challenges and stressors is becoming more and more important to both medical educators and policymakers alike, [1–3] especially in light of the rising proportions of students experiencing distress, burnout or depression during medical school [1–4] Although stress experienced by medical students can be related to a myriad of factors, a well-known stressor is related to the structure of clinical rotations in which students repeatedly transition between departments [5, 6]. During this time, students are confronted with a lot of uncertainty, for example in relation to their novice position and role within the healthcare team, patients’ emotions, and the realities of patient care within hospital cultures which are often perceived as harsh or negative [7–10].

Although stressful, these factors are a representation of what it means to work as a physician; and as such the perspective that students’ distress should also be considered an opportunity for personal and professional development has been voiced [5, 11]. The way in which individuals deal with possible stressors is called coping [12]. As Bynum et al. [11] argue, efforts to eliminate all states of impaired wellness may also eliminate opportunities to develop constructive coping mechanisms and future resilience for students. However, developing effective coping mechanisms and resilience requires formal guidance and support [13].

In recent years, medical schools have gradually responded with an increasing array of formal guidance and support by offering student wellness and support initiatives in their (undergraduate) curricula [14–17]. These initiatives aim to support 1) students’ experiential learning (i.e., learning through participation) during clinical rotations [18], and 2) personal development, i.e., stimulating the development of self-insight and healthy coping strategies to effectively deal with emotions, disturbing thoughts or unexpected situations;resulting in increased self-efficacy.

An initiative specifically aimed at aiding healthy coping mechanisms and developing a growth mindset is the implementation of a peer group reflection meeting called Intervision Meetings (IM). IM are teacher-led peer-group reflection meetings in which workplace-based learning experiences are discussed. Occurring in small groups and guided by a coach, [19] the aim of IM is to support students’ personal & professional development and autonomy by using situations experienced by students during their clinical rotations as a starting point for reflection and discussion. Not only are students stimulated to reflect on their own actions and emotions, but they are also stimulated to reflect on the experiences of others and provide feedback [20, 21].

Although similar in technique (peer groups supported by a coach) to Balint Groups [22] IM do not focus on medical content and/or the effect of patient cases on the physician like Balint groups. As such, IM borrow more strongly from Structured Group Supervision [23, 24], a tradition from social work, in which personal development issues and experiences with challenging situations are discussed and reflected upon (for more background on each method, please see Additional file 1: Appendix 1).

Research in the field of social work, [24] nursing, [25] and community practitioners [21] suggests that IM may aid in developing adequate coping mechanisms and support students’ personal and professional development [26]. However, there is still limited understanding on how and why IM contribute to students’ coping and development, especially in the undergraduate medical setting [21].

In the current study we therefore set out to evaluate how medical students participating in a three-year IM programme during their clinical rotations experienced IM and its effects on their l personal development and learning during clinical rotations.

To that end, the following research questions were formulated:

1. To what extent do students perceive that Intervision Meetings support their personal development and learning during the clinical rotations?
2. Which mechanisms do students describe that support their personal development and learning during Intervision Meetings?

## Methods

### Worldview & methodology

This study was designed from a pragmatic paradigm with the aim to make the results of the research actionable [27]. Using an explanatory mixed-methods design [28] we aimed to first capitalize on the opinions of the larger student population and thereafter go in-depth and explore some of the mechanisms influencing perceived effects of IM with a purposive sample of students.

### Context

At the start of Academic Year 2018–2019, Intervision Meetings (IM) were implemented within the Master’s in Medicine at the Faculty of Health, Medicine and Life Sciences at Maastricht University (see Additional file 2: Appendix 2 for more details on the curriculum and IM design).

The intervision programme was designed to consist of 12 meetings of 90 min each with a group of five to eight students, and scheduled during each clerkship on a university-based educational day. IM were guided by a trained coach whose role it was to create a safe atmosphere and stimulate sharing and reflection during the meetings. Coaches were either certified as coach or had a combination of a relevant professional background (physician, health professional, social scientist) with an 8-h, mandatory training. The mandatory training taught coaches how to manage group dynamics and create a safe and personal atmosphere, postpone judgement, stimulate reflection and ask questions to stimulate self-awareness.

IM were structured according to the “Incident Method”, a commonly used method for group reflection [20] ( Additional file 2: Appendix 2), which aims to ensure systematic and in-depth analysis and discussion of a case.

### Quantitative methods

An online questionnaire was developed with the aim to have the larger student population evaluate their individual experiences with IM and the perceived effect on their learning and personal development. The item-design was informed by literature on resilience, emotional intelligence, self-efficacy & coping strategies [12, 29, 30] and discussion with various experts on intervision and coaching. See Additional file 3: Appendix 3 for the specific items and their conceptual grounding. The students were asked by email to fill out the questionnaire at three time points: October 2019—April 2020, February 2021 and June 2021, and November 2021—May 2022. At those time points students had had 4 – 10 meetings. Participation was voluntary and anonymous.

### Qualitative methods

Focus groups (FGs) were chosen to get more insight into how IM affected students’ learning and development by capitalizing on the group interaction to generate data. [31] FGs were organized in August 2020, March 2021 and June 2021. The first discussion guide was informed by the same concepts as the evaluation questionnaires as well as by some of the intermediate findings coming from the evaluation questionnaire (See Additional file 4: Appendix 4). Analysis of each FG informed iterative adjustment of the discussion guide for the next group. The FGs were moderated by VvdE and observed by RES. After each FG, VvdE and RES debriefed and discussed any notes that were made. Due to the COVID-19 pandemic all FGs were online via ZOOM. To stimulate discussion in this online environment, students were repeatedly invited to react to each others’ responses and not wait for the moderator. Furthermore, the observer provided prompts to the moderator in the private chat to point out students whose body language indicated that they wanted to participate but remained silent. This resulted in active contributions by all participants. Participants were recruited through the medical students’ study association with an invitation to participate in a FG if they had had at least four meetings. The FGs were audio-recorded and transcribed verbatim by a professional transcription agency. Participation was rewarded with a pizza delivered to each individual student. FGs lasted 90 min on average each.

### Questionnaire analysis

The three questionnaires (see Additional file 3: Appendix 3) were analysed separately using descriptive statistics (N, M, SD). Separate analysis of the questionnaires was chosen because participation was anonymous and students could have theoretically filled out all three questionnaires. Open-ended questions were analysed thematically by both researchers [32].

### Focus group analysis

Data collection and analysis were iterative. All steps of the analysis process were performed by both researchers. Informed by the six steps of thematic data-analysis, [32, 33] VvdE and RES first familiarized themselves with the data, generated initial codes, searched for themes and reviewed the themes. Data and themes were compared and contrasted. Differences in interpretation were resolved through discussion until consensus was reached. Data collection seized when thematic sufficiency [34] was met.

### Reflexivity

VvdE has a PhD in communication skills training & transformational learning and is a course developer and trainer in communication and behavioural change programs at the Skillslab department of Maastricht University. When conducting this study she was coach in the IM program in the Master’s in Medicine at Maastricht University. VvdE knew none of the participants and had no professional relationship with them. RES has a PhD in medical education and is an educational scientist focusing on workplace learning and guidance. She mainly uses qualitative and mixed-methods in her research. RES conducted this study within her role as chair of task force programme-evaluation which has the purpose of monitoring and improving educational quality at the Faculty of Health, Medicine and Life Sciences. RES was not involved in the IM programme. RES knew none of the participants and had no professional relationship with them.

The purpose of the research was explained to students as being program evaluation and wanting to collect data to improve the program. Students’ open and honest sharing of their experiences and opinions was encouraged by both VvdE and RES. Confidentiality of the data was ensured to students.

## Results

### Response Questionnaire & Participation Focus Groups

A total of 357 questionnaires were filled out by students who had had four or more intervision group meetings; and respectively nine, six and four students participated in the focus group meetings (see Additional file 4: Appendix 4 for respondent and participant characteristics). All respondents (questionnaire) and participating students (focus groups) thus were either 4^th^, 5^th^ or 6^th^ year students.

#### Questionnaire results

Overall, respondents were positive about the effect experienced from attending IM (see Table 1). Respondents agreed that IM had helped them to gain insight in how they handled difficult situations during the clinical rotations, helped put things that happened in the clinical workplace in perspective, and had helped to deal more effectively with difficult situations during the clinical rotations.

**Table 1:** Questionnaire Results

	**2019–2020**			**2020–2021**			**2021–2022**		
	*Response 23.3%*			*Response 31.1%*			*Response 36.0%*		
	**N**	**M**	**SD**	**N**	**M**	**SD**	**N**	**M**	**SD**
*Intervision helped me to…*		*[1-5]*			*(1—5)*			*(1—5)*	
1. gain insight in how I am doing in my clerkships^a^	92	3.7	0.9	123	3.6	0.9	x	x	x
2. gain insight in how I handle difficult situations during the clerkships^a^	92	4.0	0.8	123	4.1	0.7	x	x	x
*a. gain self-insight*^*b*^	x	x	x	x	x	x	142	3.8	0.9
3. better deal with difficult situations during the clerkships	92	3.9	0.8	123	3.9	0.8	142	3.9	0.8
4. put things that happen in the clinical workplace into perspective	92	4.1	0.8	123	3.9	0.8	142	4.0	0.8
5. better regulate any emotions that I experience in the clinical workplace	91	3.7	0.9	123	3.6	0.8	142	3.6	0.9
6. Intervision stimulated to actively work with the insights I gained during intervision	92	3.6	0.8	123	3.6	0.9	141	3.6	0.7
7. I find the sharing of experiences with fellow students during intervision to be valuable	92	4.1	0.9	122	4.0	0.9	142	4.1	0.8
8. Intervision is a good addition to the clerkships	91	4.0	1.0	122	3.8	1.0	142	3.9	0.9
	**N**	**M**	**SD**	**N**	**M**	**SD**	**N**	**M**	**SD**
		*(1—10)*			*(1–10)*			*(1—10)*	
9. Give a grade (1–10) for the quality of the intervision programme	92	7.8	1.3	123	7.2	1.6	142	7.5	1.3

1:fully disagree, 2: disagree, 3: neutral, 4: agree, 5: fully agree

1 = lowest, 10 = highest (lower than 6 is considered insufficient)

^a^part of the 2019–2020 & 2020–2021 questionnaire, replaced by questions 'a' in the 2021–2022

^b^part of the 2021–2022 questionnaire

All respondents agreed that sharing experiences with fellow students during IM was valuable. The overall grade for satisfaction with IM fluctuated between the three points of questionnaire administration between 7 – 8 on a 10-point scale.

#### Focus group results

Three themes that described how IM impacted students coping and personal development could be constructed from the data: 1) how learning is supported through IM, 2) topics discussed during IM and putting things in perspective, and 3) conditions that need to be met in order to learn through IM.

1. How Learning Is Supported Through IM

### A safe space to learn from and with each other

First and foremost, participants described that IM provided them with an opportunity during which they felt it was safe to share their experiences and to learn how to put these experiences into perspective. Sharing their personal (workplace) experiences during IM often led to recognition in each other’s stories. This recognition resulted in feeling “not being the only one”, and enabled them to put things more easily into perspective. By being asked to discuss and analyse their experience together with the coach and peers, it also supported “perspective taking”. Being “triggered and invited” to reflect by their coach and peers, resulted in more insight into their own strengths and weaknesses, preventing doing their clinical rotations on “autopilot”.

*I always left these meetings feeling really well and that provided me with some self-assurance, like 'Ok, nice, we're not alone in this, let's go, I can do this.' (FG2 S1)*

*Often, it was really an eyeopener, like 'wow, I would have never thought of it like that' (FG2 S2)*

*It is, I think, a structural moment of reflection, so you are being forced, well, forced sounds so negative, but you get a handhold to think about it. I think that otherwise you could just coast through the clinical rotations on autopilot. (FG3 S1)*The safe atmosphere during IM also helped them to not hide potential vulnerability. This experience was often opposite to their experiences in the clinical workplace where students described how a commonly used strategy to “survive” hierarchy or an unsafe workplace culture was to hide one's vulnerability and keep up appearances.

*Sometimes in the workplace, I don't know whether you [other participants in the focus group] have the same experience, but that other students can give you the impression like everything is great, as if really everything is going well and as if everything is perfect and then I sometimes think 'no, that isn't true'. (...) during Intervision you can just be honest and then you see like 'okay, no, not everyone thinks everything is great and wonderful and pretty and fun'. (FG1 S1)*

### Vicarious learning

Students described how hearing about the experiences of peers had a “spill over effect”: participants would recognize similar situations during their own clinical rotations more easily and felt better prepared to deal with them. Hearing different alternative ways of thinking or behaving increased students’ own coping repertoire and for some participants even increased their self-confidence.

*Maybe it has also made me more self-confident, because those tips can, for example, help you change your behaviour,... that would be something you had heard during Intervision, like 'oh, right, and this is how my peer addressed it, maybe I should try that out for myself'. And if that works well, then you yourself also grow* [as a person in your learning process]. (FG3 S2)

*So you could say that all those other perspectives did teach me to think differently and also, during a next time, to deal with things differently. (FG1 S2)*

2. Topics discussed during im and putting things in perspective

The most frequently mentioned topics that were discussed during IM were coping with stress, heavy workload and uncertainty; how to keep a healthy work-life balance; how to deal with unpleasant behaviour or negative feedback from a supervisor; how to deal with patients’ suffering and death; how to deal with unwanted behaviour or unsafe situations; and how to make career choices. As mentioned above, IM added value by exchanging concrete tips on how to deal with these topics.

But next to that, participants also explicitly mentioned the added value of hearing from their coach that “something wasn’t their fault”. This enabled them to deal more effectively with situations outside their locus of control. Therefore, students felt that IM helped them to on the one hand learn to accept and let go and take things less personally, while on the other hand it helped them to keep a healthy frame of reference on what is normal and acceptable and what is not.

*Some creation of awareness of emotions that you yourself have and also how your behaviour is partially* *influenced by these (…) and that you learn to become aware of it while it is happening. I think that* [achieving that level of awareness]* really helps you to take everything less personal. (FG3 S2).*

*But sometimes the answer was 'yes, well, you cannot change it and this situation is not caused by you'. That was sometimes just really nice to hear. (FG1 S3)*

3. Conditions That Need To Be Met In Order To Learn Through IM

### Safety is key

As a first condition, the IM group needed to feel safe for each of the participants in order for them to show their vulnerability, or dare to share. Group size (max. 5 – 8 students) and the coach played an important role in establishing this safety by ensuring that the discussed issues did not “leave the room”, but also by stimulating a non-judgemental attitude and creating a non-judgemental atmosphere. Students appreciated it when coaches also showed their own vulnerability. The fact that assessment did not play a role in IM added significantly to perceived safety.

*Yes, and also, in the clinical rotations there are always people walking around that need to assess you and you just don't want to say everything. In the intervision environment anything can be said*. (FG1 S4)

### The coach

All participants felt the role of the coach was crucial: it could make or break the IM experience. However, structuring the IM meetings was not self-evident. When the coach followed the Incident method too strictly, participants indicated that this often “killed” the discussion. When the coach left the discussion flow too freely, this was also perceived as ineffective.

*And because you had to follow that structure, many important moments were passed by or lost, I think. (FG1 S4)*

*In the beginning the coach really tried to gain depth of discussion by endlessly probing and then you saw people just losing the will to talk about it at all. So then there were these awkward silences and it turned into just sitting there till time was up. (FG2 S4)*

*…that too little is done with the situation, that there are too few tips, concrete points for attention. That it is just a lot of complaining without reflection and then the real effect stays absent. (FG3 S2)*

Students indicated that the professional background of the coach was less relevant. Mastering the skill of paying sincere attention, showing empathy, and being able to ‘ask the right questions at the right moment’, were perceived to be more important to students than whether the coach had a medical background or not.

*I do think that a non-medical background as coach has advantages….in this way you really focus on the emotional and communicational aspects, instead of falling into the pitfall of medical coaches who tend to “switch more easily to the medical content” .(FG1 S5)*

Finally, good coaches were able to transform the introduced case to a meaningful learning experience for the entire group. Participants did note that students’ own ability to reflect played a role in this regard. The more this ability was present among the students and/or the more this ability was fuelled and nurtured/fed by the coach, the more effective the IMs were perceived.

*I do think that intervision and reflection is a learned skill and that you can learn to see vulnerability as a strength, but also to give feedback to others and that that is also an added value of intervision, that you learn to do this with fellow doctors or at least share this with future fellow doctors. (FG3 S1)*

## Discussion

As calls have increased for providing guidance to students in how to cope with the potential stress resulting from medical school training, this study sought to evaluate how undergraduate medical students going through their clinical rotations experienced the effect of a three-year programme of IM on their learning and personal development. Using an explanatory mixed-methods approach, the results from this study highlight that IM were perceived to benefit students’ ability to cope with challenging situations during clinical rotations. Focus group discussions pointed to how IM supported students to develop their coping and highlighted the key role of the coach in this process.

Our work resonates with the positive effects of IM on its participants reported elsewhere, [21, 24–26] strengthening the suggestion that implementing IM for medical students going through clinical rotations may be beneficial. Participants in this study described how IM created an increase in self-awareness by active self-reflection supported by peers and the coach. Sharing and recognizing each other’s’ situations, stories or problems; as well as hearing alternative ways of coping, helped students to put things into perspective, try out alternative behaviour, and develop self-efficacy regarding dealing with challenging situations in their clerkships. These results suggest that introducing IM will equip students going through clinical rotations with a combination of skills that will help them approach challenges as learning opportunities and trust that they will be able to manage challenging workplace learning situations in the future [11]. Longitudinal, follow-up research needs to confirm to what extent the skills learned during IM persist into residency training and beyond.

Our study suggests that successful implementation of IM is dependent on several factors. The role of the coach was reported to be essential in this regard. Our results describe several tasks for the coach in order to make IM effective: create a safe atmosphere, strike the right balance between a structured and more open approach to meetings, and ask the right questions with the right amount of depth to ensure that the introduced case is transformed into a meaningful learning experience for the whole group. This shows that being an IM-coach requires a unique set of skills, [35] akin to that of a mentor [3, 36, 37], in order to assist in personal, social and professional development. Our results could provide direction to both practical training of future IM-coaches as well as provide guidance to future research aimed at further exploring the IM-coach role. A stable group composition could be considered favourable for creating a safe atmosphere. However, due to logistical complications in our setting, the students had to change groups for the final two meetings. Future research needs to explore how this impacts experienced safety by students.

It should be noted that participants in our study addressed the juxtaposition of what they experienced during IM versus in the clinical workplace regarding vulnerability. During IM students felt safe to share, whereas in the clinical workplace students felt compelled to keep up appearances to “survive”. If we want our students to develop healthy coping mechanisms, solutions should not only be sought in focusing on the individual. Students might perceive efforts aimed at building individual resilience as futile without changes in professional values and sustained organizational support; as our and other studies have shown [38]. Future research could therefore focus on how individual and organizational solutions might be combined to deliver even greater improvements in student (or physician) well-being than those achieved with individual solutions only [39].

There are some limitations to our research. First, the response rate to our questionnaire was relatively low. In addition, due to the anonymous nature of the questionnaire administration, it is possible that each sample consists of similar or different students making it difficult to compare the samples and inviting response bias. We cannot say whether the same students filled out the questionnaire at all three time points or that these were different students. As such, we cannot say anything about the extent to which these ratings will be stable over time. Furthermore, since the purpose of our research was program evaluation and not instrument development and validation, we did not perform separate psychometric validation of the questionnaire. However, due to the longitudinal and mixed methods nature of our research design, we aimed to provide a general but rich evaluation of the IM method. Future research should also incorporate the perspective of other stakeholders like coaches and eventually alumni to get a better understanding of the conditions under which IM is most beneficial, in the short term as well as the long term. Second, due to the pandemic we had to perform the Focus Group Discussion via ZOOM. We tried to mimic the focus group experience to the best of our possibilities but cannot be certain that live focus group discussion would have yielded even more insight in the students’ experiences. Third, peer group reflection meetings have been studied under various different names. Although IM is informed by the traditions of Balint groups and Structured Group Supervision, it is in essence very similar to Structured Group Supervision (also see Additional file 1: Appendix 1). However, to avoid confusion with workplace supervision, a dominant concept in medical education, we have chosen to label this educational format as Intervision Meetings. Research and practice on peer group reflection meetings may therefore be further complicated by the variety of terms used to describe them [24]. By clearly defining how we operationalized IM within our context, linking it to other definitions and providing an in-depth description of how the method was used in our curriculum, we hope to have increased the transferability of this research to other educational contexts.

In sum, participants in our study felt that a three-year IM programme during clinical rotations aided them in transforming the distress caused by clinical rotations to opportunities for personal and professional development. Regular meetings, supported by an effective coach and with sufficient depth of discussion were considered pre-conditions for success. Medical students’ self-efficacy is, among others, dependent on their ability to effectively cope with distress. IM is a potential method medical schools can use to aid their students on their journey of personal and professional development.

## Supplementary Information

**Additional file 1.** 

## Data Availability

The datasets used and/or analysed during the current study are available from the corresponding author on reasonable request.
