# Supplementary material for: How do peer group reflection meetings support medical students’ learning and personal development during clinical rotations?
Source: BMC Med Educ. 2023 Jul 6;23:499. doi: 10.1186/s12909-023-04481-0 (PMC10327380; doi:10.1186/s12909-023-04481-0)
Supplement: Supplementary file 1 — Additional file 1. [file 12909_2023_4481_MOESM1_ESM.docx]

**Appendix 1**

**Definition of other, common Peer Group Reflection Formats**

Other, well-known reflective group methods that have been elaborately described in the literature are Balint Groups (Ruth 2009) and Structured Group Supervision (Akhurst and Kelly 2006, Wilbur 2008).

Balint Groups originate from the field of General Practice and specifically focus on the relationship between the patient and the physician as well as the medical content of the patient cases (Ruth 2009).

Structured Group Supervision originates from the field of Social Work and focuses more on personal development and learning experiences during workplace learning (Akhurs and Kelly, 2006).

These Peer Group Reflection meetings have the guidance by a coach in common, as well as focusing on issues brought in by individual peers and reflecting on them as a group.

Intervision Meetings follow the same set-up and structure as Structured Group Supervision, however, the word ‘supervision’ has a different connotation within the field of medical education, i.e., supervising residents during clinical practice. Therefore we chose to adhere to the most commonly used term for these group reflection meetings within the medical field: *Intervision* meetings.

**Appendix 2**

**Context of the research**

*Master of Medicine at the Faculty of Health, Medicine and Life Sciences at Maastricht University, Maastricht, the Netherlands*

Following a three-year, pre-clinical curriculum designed around principles of problem-based learning, the Master’s in Medicine consists of three clinical years in which students rotate through integrated clerkships of various duration and varying focus (e.g. Internal Medicine, Surgery, Gynaecology and Paediatrics, Family Medicine & Social Medicine).

*Programmatic Assessment*

The curriculum is competency-based and supported by programmatic assessment and a mentor for each enrolled student. Each mentor has to assess the portfolio of his or her students. This in contrast to the role of the coach during the Intervision meetings, in which the coach does not have an assessing role. In this regard there was no power dynamic between the coaches and the students during IM. This worked positively in creating an atmosphere of safety for students to feel free and dare to bring up issues or problems.

*Structure of Intervision Meetings*

The aim of IM was to discuss cases (experiences, thoughts, emotions) put forward by students. These cases were discussed in order to distil meaningful points for learning and development for each of the group members. IM were organized during ‘educational days’ in the clerkship programme. During these educational days, students are not in the clinic and receive general skills training and lectures on basic sciences and ethics. Timing of the meetings differed according to the students’ clerkship scheduling track.

IM groups consisted of 5 to 8 students who stayed together for the first 10 meetings. Scheduling conflicts requires reassignment of students and coaches for the final two meetings. This was explained to the students and also explained as an opportunity for them to practice participation in a new IM group composition, similarly to what is expected of them during their future career life.

Participation at the IM was not obligatory. There were no situations reported where students did not want to participate, except when students were ill or had a good reason to be absent.

*The Intervision Coach*

The coach was responsible to manage the structure of the meetings in agreement with all participating students. The cases brought in by the students were leading and groups could choose which structure worked best for them; either decide on forehand who wanted to bring in a case, or decide during the meeting itself who had a case to bring in.

To be able to fulfil the role of coach, either a qualification as certified coach or a combination of a relevant professional background (physician, health professional, social scientist) with intrinsic motivation to coach and attending an 8-hour training spread over 2 days were mandatory. The content of this training covered skills in creating a safe learning climate, group dynamics, stimulating in-depth discussion and reflection, dealing with resistance, etc.

**Incident Method** (Bellersen & Kohlmann 2019)

Step 1: The participant (the problem owner) introduces the theme, problem or challenge they would like to discuss

Step 2: Other participants ask questions to further explore and clarify the topic of discussion

Step 3: Other participants suggest other ways of dealing with the topic (brainstorm)

Step 4: Other participants make a short summary of the brainstorm and formulate some suggestions

Step 5: The problem owner provides their response - what have I heard, what will I take with me? How will I translate this to concrete intentions and behaviour.

**Appendix 3**

**Questionnaire Items and Conceptual Grounding**

| **Items** | **Conceptual Grounding** |
| --- | --- |
| *Intervision helped me to...§* |  |
| 1. Gain insight in how I am doing in my clerkships* | Emotional Intelligence |
| 2. Gain insight in how I handle difficult situations during the clerkships | Coping Strategies |
| a. gain self-insight** |  |
| 3. Better deal with difficult situations during the clerkships | Self-Efficacy |
| 4. Put things that happen in the clinical workplace into perspective | Resilience |
| 5. Better regulate any emotions that I experience in the clinical workplace | Emotional Intelligence |
|  |  |
| 6. Intervision stimulated to actively work with the insights I gained during intervision | Self-Efficacy |
| ‍7. I find the sharing of experiences with fellow students during intervision to be valuable | Coping Strategies |
| ‍8. Intervision is a good addition to the clerkships | Experiential Learning |
| ‍9. Give a grade (1-10) for the quality of the intervision programme | General quality indicator |
| ‍  § Items 1 – 8 have a Likert Scale, 1; fully disagree, 2; disagree, 3; neutral, 4; agree, 5; fully agree  Item 9: 1 lowest, 10 = highest (lower than 6 is considered insufficient)  * part of the 2019-2020 & 2020-2021 questionnaire, replaced by question ‘a’ in the 2021-2022 questionnaire  ** part of the 2021-2022 questionnaire | |

**Focus Group Discussion Guide**

| **Topic** | **Questions** |
| --- | --- |
| Could you please introduce yourself | - Which clerkship are you currently participating in? - Which year of training are you in? - How many IM did you attend thus far? |
| Added value IM | How did you experience the IM?  What was the added value of IM to you?  Under what conditions did IM work for you? |
| Emotional experiences | To what extent did participating in IM help you in dealing with emotional experiences?  What type of situations were these mostly?  Were there certain recurring topics that were discussed? |
| Coping strategies | To what extent did IM help you to gain insight in the ways in which you deal with difficult situations?  To what extent did IM help you to develop alternative ways to deal with difficult situations? |
| Resilience | To what extent has participating in IM helped you to   - put things into perspective? - be more assertive? - pay more attention to restore and recover? |
| Additional points of attention for interviewer (resulting from questionnaire and focus group analysis) | - During which moments did IM help you most? - Which types of (difficult) situations did you discuss during IM? Did you also discuss difficult patient cases? - What is the role of the workplace versus IM in dealing with difficult situations? Would you rather discuss the things you discuss during IM at the workplace? - Did you experience discussing something during IM and thereafter recognizing it in the workplace? - Do you recognize the added value of IM in becoming more aware of yourself? - When looking back, how did you experience IM in the beginning and how do you experience it now? Does that differ? Why? - To what extent do you feel that IM is useful for all students? Why(not)? - To what extent do you think that IM will help prevent problems in future? - Were the topics during IM discussed deeply enough? |
| Ending Question | Is there something you would like to add/share? Any topics that remained undiscussed? |

**Appendix 4**

*Response Characteristics Questionnaires*

|  | Response N (%) | N meetings attended by respondents at time of questionnaire |
| --- | --- | --- |
| Questionnaire 1 | N = 92 (23.3%) | 4 meetings 23.9%  5 meetings 41.3%  6 meetings 34.8% |
| Questionnaire 2 | N = 123 (31.1%) | 4 meetings 14.8%  5 meetings 13.9%  6 meetings 13.9%  7 meetings 13.1%  8 meetings 16.4%  9 meetings 7.4%  10 meetings 20.5% |
| Questionnaire 3 | N = 157 (36%) | 10 meetings, 100% |

*Participants Characteristics Focus Groups*

|  | N | N intervision meetings completed at time of FG | F/M |
| --- | --- | --- | --- |
| Focus Group 1 | 9 | 10, 5, 6, 10, 10, 8, 9, 10, 8 | 6 female 3 male |
| Focus Group 2 | 6 | 10, 10, 9, 10, 4, 10 | 5 female 1 male |
| Focus Group 3 | 4 | 4, 7, 10, 4 | 2 female 2 male |
